# With computed tomography confirmed anterolateral left ventricular pseudoaneurysm in patient with dilatative alcoholic cardiomyopathy

**DOI:** 10.2478/v10019-011-0021-8

**Published:** 2011-07-20

**Authors:** Mitja Letonja, Marija Santl Letonja

**Affiliations:** 1 General Hospital Ptuj, Medical Faculty University of Maribor, Maribor, Slovenia; 2 General Hospital Murska Sobota, Murska Sobota, Slovenia

**Keywords:** left ventricular pseudoaneurysm, echocardiography, computed tomography

## Abstract

**Background:**

Pseudoaneurysms are rare complications of myocardial infarction with propensity for rupture. There is still a challenge with which diagnostic imaging we performed a final diagnosis of pseudoaneurysm and differentiate it from true aneurysm what is clinically important due to the different treatment.

**Case report.:**

We presented the unusual case of a 56-year-old man with signs of decompensated heart failure which had worsened a few months before hospitalization. We believed that during worsening of symptoms the patient suffered a silent myocardial infarction complicated by subacute free wall rupture which resulted into left ventricular pseudoaneurysm formation without tamponade. Echocardiography showed dilatative cardiomyopathy which was already present years before and a very rare location of the left ventricular pseudoaneurysm on the anterolateral part of the left ventricle. Pseudoaneurysm was confirmed with CT scan. Due to the severity of contractile dysfunction and no response in treatment for congestive heart failure the directive for the resection was tempered and the patient died due to the progressive heart failure and embolic phenomena.

**Conclusions:**

This report shows the importance of non-invasive imaging diagnostic evaluation of acute decompensated heart failure where echocardiography and chest X-ray are the first diagnostic steps. Based on those findings further imaging diagnostic steps must be performed such as CT scan in our case which finally confirms left ventricular pseudoaneurysm with dilatative cardiomyopathy.

## Introduction

Majority of patients with the acute free wall rupture after the myocardial infarction die suddenly, but only a few of them developed ventricular pseudoaneurysm mostly when the rupture is subacute and confined by epicardium, pericardial adhesions and thrombus formation.^1^,^2^ Pseudoaneurysm can be developed in a few days after a myocardial infarction or even two years later.^3^ Pseudoaneurysm of the left ventricle (PSALV) after a silent myocardial infarction is very rare. Most frequently reported clinical symptoms of pseudoaneurysm are a heart failure (reported from 36 to 70%), a chest pain (30%) and a dyspnea (25%).^4^,^5^ Only 10–13% of patients with pseudoaneurysm are asymptomatic. Pseudoaneurysm has propensity for a rupture with a fatal outcome in about half of the cases.^6^ Pseudoaneurysms are usually located on the posterolateral or upper lateral site of the left ventricle but usually not in the anterolateral region as in our case.^2^,^7^,^8^

Diagnosis of PSALV is mostly confirmed with ventriculography, echocardiography, pulse Doppler, computed tomography (CT), and recently with magnetic resonance imaging (MRI).^9^–^14^ We confirmed the diagnosis of a PSALV with echocardiography and CT scan that are very effective imaging methods for accurate diagnoses of cardiovascular anomalies.^13^–^16^

## Case report

We present a pseudoaneurysm in the anterolateral part of the left ventricle in a patient with dilated alcoholic cardiomyopathy who suffered a heart failure a few months ago after a silent myocardial infarction. Our patient was a 56-year-old man who was an alcoholic and his general practitioner reported a repeated abuse of alcohol despite of the psychiatric treatment. We observed a dilated cardiomyopathy for 5 years before he was admitted to our hospital and it had also been confirmed that he had hepatic cirrhosis Child A 7 years before he was admitted to our hospital. He had been treated for heart failure with the ACE inhibitor and spironol-actone but he did not take his medication regularly. We conclude that dilatative cardiomyopathy and hepatic cirrhosis was due the alcohol abuse. Eight months prior the patient’s admittance in hospital, the main finding of the echocardiography was a dilated cardiomyopathy with a severely reduced ejection fraction, but without o segmental loss of contractility or aneurysm of the left ventricle. He was also a smoker.

At admission to our hospital the patient complained about progressive dyspnoea, syncope, confusion and 10 kg weight loss during previous 2 months. At physical examination the patient was found to be confused, he had cachexia (56 kg) and jaundice, with blood pressure of 120/95, a regular pulse of 125 beats/min, respiratory rate 27 breaths/min. He had a jugular venous distension to the angle of the jaw at 30 degrees. The chest examination showed dullness on percussion in the basal part of the lungs. The cardiac examination was unremarkable. The liver was enlarged and there was peripheral oedema.

**FIGURE 1:**
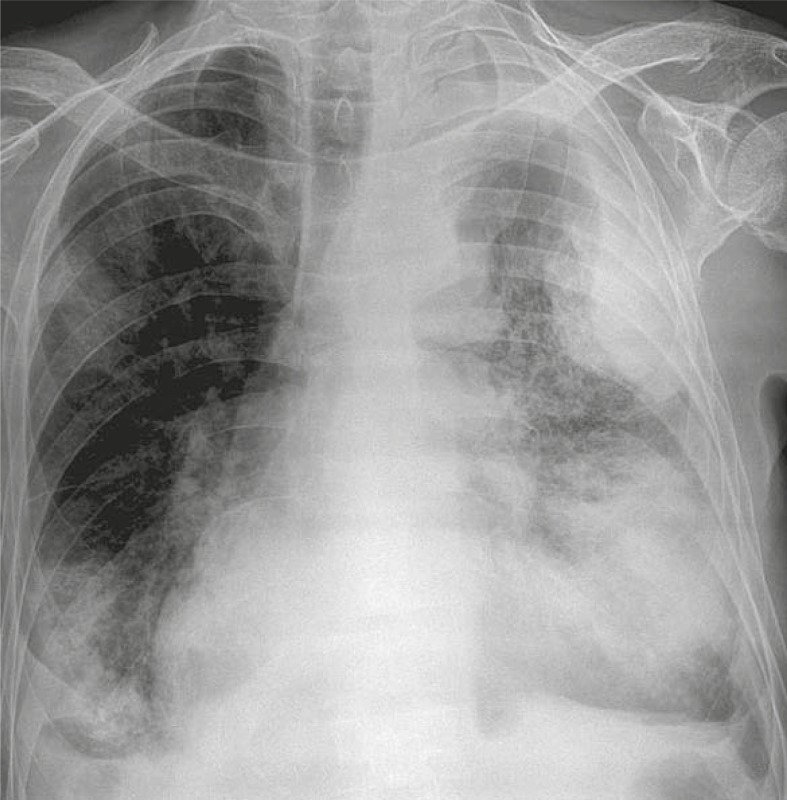
Frontal radiograph shows a bulge projected from anterolateral aspect of the ventricle and pleural effusion.

**FIGURE 2:**
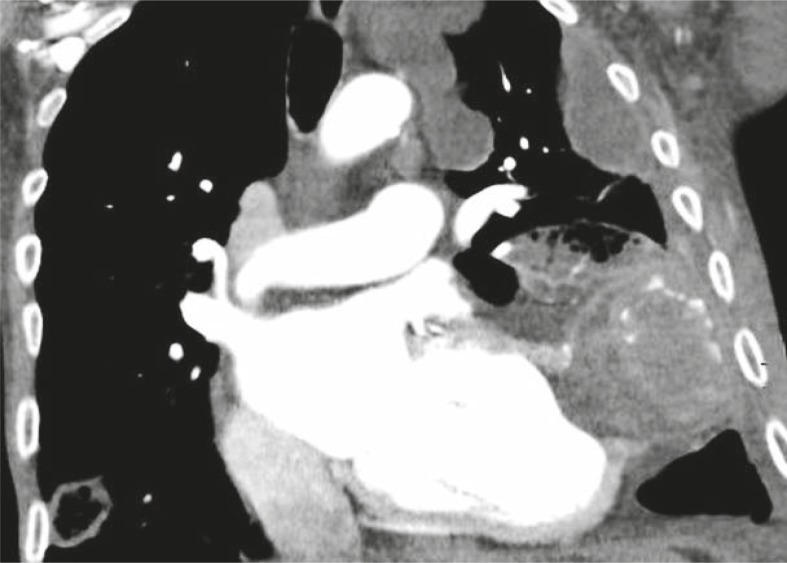
CT image shows a neck created by the orifice of the ruptured myocardium and a large thrombus occupying pseudoaneurysmal cavity.

On hospital day 1 we performed haematological tests which were normal except mild makrocytosis (MCV 97 fl). Liver function tests were pathological (total bilirubin 264 μmol/L, direct bilirubin 208 μmol/L, sAST - serum aspartate aminotransferase 1,22 μkat/L, sALT – serum alanin aminotransferase 2,58 μkat/L, γGT - gama glutamiltranspeptidase 0,59 μkat/L, alkaline phosphatase 2,14 μkat/L), he had hyperammoniemia (194 μmol/L) and INR were prolonged (1,68). The levels of cardiac enzymes and troponine were normal. N-terminal pro-B-type natriuretic peptide (NT-proBNP) was highly elevated (24357 ng/L). EKG showed sinus tachycardia 128 beats/min and QS from V1 to V6 with non-specific mild elevation of ST segment. Chest X-ray showed a large soft-tissue mass contiguous with the apical aspect of the heart (Figure 1). We concluded that the patient had the symptoms of hepatic coma and fluid retention due the heart failure. We started treatment with lactulose and furosemid. We also increased the dose of ACE inhibitor and added carvedilol. Hyperammoniemia was resolved with a treatment and the patient became orientated after 3 days. Echocardiography demonstrated an enlarged diastolic volume of the left ventricle (183 ml) with a severely depressed ejection fraction (the left ventricular volume and the ejection fraction were calculated on 10% using bi-plane Simpson’s rule). In the apex there was an abrupt interruption of the left ventricular wall, constituting a narrow necked communication between the left ventricular cavity and the pseudoaneurysm, which contains thrombus. We also performed abdominal ultrasound which showed an increase in the echogenicity of the liver and distended hepatic veins consistent with an alcohol-induced liver disease and a heart failure. After 5 days we repeated chest X-ray which showed no difference according to the previous one. CT was performed for further diagnostic step using sagittal and axial cine mode images and showed more precisely a pseudoaneurysm with the site of cardiac rupture. The size of pseudoaneurysm with orifice measured 45 mm and a cavity of the pseudoaneurysm was larger than orifice and measured 75 mm, including a large thrombus measured 55 mm in length and 70 mm in width (Figure 2). Cardiac Surgery Centre was consulted, but due to the low ejection fraction and no response on heart failure therapy the directive for the resection was tempered. We increased a dose of low molecular heparin and added dobutamine but despite that, progressive heart failure and embolic phenomenon resulted in death.

The pathology report confirmed the presence of marked variations in myocyte size; some myocardial cells were hypertrophied and others were atrophied. There were areas of interstitial and perivascular fibrosis. The report was consistent with the dilated cardiomyopathy. Atherosclerosis was found particularly in the left anterior descending (LAD) coronary artery without thrombosis. Apical aneurysm included only fibrous tissue, pericardium and no myocardial cells consistent with a pseudoaneurysm. There were embolic masses in pulmonary circulation beside congestion of the lung.

## Discussion

The PSALV is a rare complication of myocardial infarction in which a ventricular free wall rupture at necrosis site and escape of blood are locally contained by adherent pericardium shaping the aneurysmatic sac. The sac progressively enlarges and its internal surface is covered by thrombus. Less often PSALV has been reported in association with cardiac trauma, myocarditis, infective endocarditis, cardiac surgery, rheumatic fever, syphilis and tuberculosis.^17^

PSALV is usually presented with non-specific symptoms as in our case where the patient complained of dyspnoea, syncope, confusion and weight loss. On admission the patient had clinical manifestation of heart failure and also plasma N-terminal pro-brain natriuretic peptide (NT-proBNP) confirmed the diagnosis of heart failure. We also observed Q waves and ST segment changes which are common although non-specific signs of PSALV. Chest X-ray abnormalities are present with abnormal cardiac contour or cardiomegaly in more than 95% of patients. We also observed a large soft-tissue mass contiguous with the apical aspect of the heart (Figure 1). The enlargement of the aneurysm on sequential radiographs is a characteristic feature of false aneurysm observed in more than 50% of patients.^17^ The initial findings of mass on chest X-ray and non-specific electrocadiographic changes require further diagnostic steps and transthoracic echocardiography is usually the first imaging modality used for this purpose.

The most characteristic echocardiographic criteria of PSALV which were also confirmed in our patient include: the saccular space outside the left ventricle, break in the contour of the myocardium, an orifice diameter smaller than the cavity of the pseudoaneurysm and the presence of thrombus in the internal wall of the aneurysmatic sac. The communication between the ventricle and the cavity differentiates the pseudoaneurysm from localized pericardial effusion or haematoma, and a pericardial cyst.^7^,^10^ The major limitation of transthoracic echo is a limited acoustic window so that the pseudoaneurysm orifice could not be accessible to the ultrasound beam.

Although the diagnosis of PSALV is established by transthoracic echocardiography, further imaging with CT or MRI should be performed because pericardial mass can usually not be differentiated with echocardiography as a true or false aneurysm. The difference between true and false aneurysms is clinically important because false aneurysms have propensity for rupture.^1^,^2^,^6^ With the CT scan a more distinct depiction of the site of the cardiac rupture, the extent of the rupture, the size and location of PSALV are showed (Figure 2). The same information can be obtained with the MRI. As in our case, the histological examination finally confirms the diagnosis of the PSALV where the wall of the false aneurysm has no remnants of myocardial tissue which is the major distinction to true aneurysms.

Most reports considered surgery as the appropriate treatment for PSALV since untreated pseudoaneurysms have an approximately 30% to 45% risk of rupture. Mortality rates are in patients who underwent surgery from 19% to 35% what was significantly lower than in those who were treated medically (48% to 55%). Operative mortality in most reports is related to the severity of the contractile dysfunction in the reminder of the ventricle.^4^,^5^

In our patient physical findings and laboratory evidence of a liver failure (confusion due hyperamoniemia, abnormal prothrombin time and hypoalbuminemia in addition to elevated aminotransferases) with a documented history of alcohol abuse were consistent with the diagnosis of the alcoholic liver disease. The liver disease was combined with dysfunction of heart due to dilated alcoholic cardiomyopathy and coronary artery disease which was complicated with PSALV.^16^ The most possible scenario concerning our patient was that the anterolateral myocardial infarction was complicated by the subacute free wall rupture which resulted into pseudoaneurysm formation without tamponade. Although the time of acute necrosis or the moment of cardiac rupture cannot be precisely determined it must have occurred after the echocardiography, which had been done eight months before the patient was admitted to our hospital, (during the echocardiography we did not observe PSALV) and before the clinical deterioration which was reported two months before hospitalisation. Because of pre-existing dilated cardiomyopathy due to alcohol abuse with a severe reduced ejection fraction PSALV did not rupture for quite a long period of time. Dilated cardiomyopathy also enabled the survival of rare aneurism in the anterolateral surface of the left ventricle which usually leads to haemopericardium and death. Besides the normal value of cardiac necrosis enzymes and the ECG changes did not reveal recent acute transmural necrosis. The existence of large laminated thrombus makes us speculate that acute necrosis and cardiac rupture occurred a long time before the admission. Because of no response in drug therapy for congestive heart failure in our patient the directive for the resection was tempered and the patient died due to the progressive heart failure and embolic phenomena.

## Conclusions

This report highlights the importance of basic non-invasive imaging diagnostic evaluation (echocardiography and chest X-ray) of the acute decompensated heart failure and afterwards the decision for further non-invasive imaging modalities or invasive cardiologic imaging. The case shows that we cannot presume the diversity or combination of the clinical picture based on the clinical ground. CT scan finally differentiates left ventricular pseudoaneurysm from true aneurysm as the complication of a silent myocardial infarction in our patient with alcoholic dilatative cardiomyopathy.

## References

[1] Dachman AH, Spindola-Franco H, Solomon N (1981). Left ventricular pseudoaneurysm. Its recognition and significance. JAMA.

[2] Martin RH, Almond CH, Saab S, Watnon LE (1997). True and false aneurysms of the left ventricle following myocardial infarction. Am J Med.

[3] Davidson KH, Paris A, Harrington J, Barsamian E, Fischbein M (1977). Pseudoaneurysm of the left ventricle: an unusual echocardiographic presentation. Review of the literature. An Intern Med.

[4] Frances C, Romero A, Grady D (1998). Left ventricular pseudoaneurysm. J Am Coll Cardiol.

[5] Eren E, Bozbuga N, Toker EM, Keles C, Rabus MB, Yildirim O (2007). Surgical treatment of post-infarction left ventricular pseudoaneurysm. Tex Heart Inst J.

[6] Zoffoli G, Mangino D, Venturini A, Terrini A, Asta A, Zanchettin C (2009). Diagnosing left ventricular aneurysm from psudo-aneurysm: a case report and a review in literature. J Cardiothorac Surg.

[7] Stoddard MF, Dawkins RP, Longaker RA, Shih A (1993). Transesophageal echocardiography in the detection of left ventricular pseudoaneurysm. Am Heart J.

[8] Raquel M, Tegtmeier T, Smith SA, Ognibene A (1997). Left ventricular pseudoaneurysm presenting twenty-eight months after myocardial infarction. Angiology.

[9] Tuan J, Kaivani F, Fewins H (2008). Left ventricular pseudoaneurysm. Eur J Echocardiogr.

[10] Cho MN, Mehta SK, Matulevicius S, Weinstein D, Wait MA, McGuire DK (2006). Differentiating true versus pseudo left ventricular aneurysm. A case report and review of diagnostic strategies. Cardiol Rev.

[11] Harrity P, Patel A, Bianco J, Subramanian R (1991). Improved diagnosis and characterization of postinfarction left ventricular psudoaneurysm by cardiac magnetic resonance imaging. Clin Cardiol.

[12] Prakash S, Garg N, Xie GY, Dellsperger KC (2010). Giant left ventricular pseudoaneurysm. J Cardiovasc Comput Tomogr.

[13] Beslic S, Beslic N, Beslic S, Sofic A, Ibralic M, Karovic J (2010). Diagnostic imaging of traumatic. pseudoaneurysm of the thoracic aorta. Radiol Oncol.

[14] Kim MN, Park SM, Kim SW, Lee KN, Kim JS, Kang EJ (2010). Progression of left ventricular pseudoaneurysm after an acute myocardial infarction. J Cardiovasc Ultrasound.

[15] Mukhopadhayay S, Yusuf J, Mehta V, Nathani S, Goyal V (2010). Pseudoaneurysm of the left ventricle in a young asymptomatic female. Echocardiography.

[16] Gjikolli B, Hadzihasanovic B, Jaganjac S, Herceglija E, Niksic M, Hadzimehmedagic A (2008). Treatment of complicated case with subclavia steal syndrome and stenosis of common iliac artery. Radiol Oncol.

[17] Higgins BC, Lipton MJ, Johnson AD, Peterson KL, Vieweg WVR (1978). False aneurysms of the left ventricle. Radiology.

[18] Masani F, Kato H, Sasagawa Y (1990). An echocardiographic study of alcoholic cardiomyophaty after total abstinence. J Cardiol.

